# Framework of Collagen Type I – Vasoactive Vessels Structuring Invariant Geometric Attractor in Cancer Tissues: Insight into Biological Magnetic Field

**DOI:** 10.1371/journal.pone.0004506

**Published:** 2009-02-18

**Authors:** Jairo A. Díaz, Mauricio F. Murillo, Natalia A. Jaramillo

**Affiliations:** Department of Pathology, Medicine School, Laboratory of Pathology, Clinical Corporation, University Cooperative of Colombia, Villavicencio, Meta. Colombia; Health Canada, Canada

## Abstract

In a previous research, we have described and documented self-assembly of geometric triangular chiral hexagon crystal-like complex organizations (GTCHC) in human pathological tissues. This article documents and gathers insights into the magnetic field in cancer tissues and also how it generates an invariant functional geometric attractor constituted for collider partners in their entangled environment. The need to identify this hierarquic attractor was born out of the concern to understand how the vascular net of these complexes are organized, and to determine if the spiral vascular subpatterns observed adjacent to GTCHC complexes and their assembly are interrelational. The study focuses on cancer tissues and all the macroscopic and microscopic material in which GTCHC complexes are identified, which have been overlooked so far, and are rigorously revised. This revision follows the same parameters that were established in the initial phase of the investigation, but with a new item: the visualization and documentation of external dorsal serous vascular bed areas in spatial correlation with the localization of GTCHC complexes inside the tumors. Following the standard of the electro-optical collision model, we were able to reproduce and replicate collider patterns, that is, pairs of left and right hand spin-spiraled subpatterns, associated with the orientation of the spinning process that can be an expansion or contraction disposition of light particles. Agreement between this model and tumor data is surprisingly close; electromagnetic spiral patterns generated were identical at the spiral vascular arrangement in connection with GTCHC complexes in malignant tumors. These findings suggest that the framework of collagen type 1 – vasoactive vessels that structure geometric attractors in cancer tissues with invariant morphology sets generate collider partners in their magnetic domain with opposite biological behavior. If these principles are incorporated into nanomaterial, biomedical devices, and engineered tissues, new therapeutic strategies could be developed for cancer treatment.

## Introduction

In previous research, we have described and documented self-assembly of geometric triangular chiral hexagon crystal-like complex organizations (GTCHC) in human pathological tissues. The architectural geometric expression was described on macroscopic and microscopic levels mainly in cancer processes. On the basis of the electro-optical model, research has demonstrated that molecular crystals are represented by triangular chiral hexagons. These triangular chiral hexagons are derived from collision events against collagen type I fibrils, emerging at microscopic and macroscopic scales in the lateral assembly of each side of the hypertrophy of the helicoid fibers. The helicoids fibers represent flow of energy in hierarchically cooperative chiral electromagnetic interaction in pathological tissues, and arise as geometry of the equilibrium in perturbed biological systems. [1]

In this article we have documented and gathered insights into the magnetic field in cancer tissues and how it generates a functional geometric attractor complex in their entangled environment. This geometry occurs on documented collider partners, that is, pairs of spiral subpatterns twisted in opposite directions that generate in this rotational movement powerful electromagnetic forces. These forces are exerted over collagen type 1 fibrils and influence the dipole behavior of vascular cells. Centrifugal expansion happens when the axis spins in one direction and contraction happens when centripetal forces spin in the opposite direction. This causal effect evolves onto collagen-vascular framework that surges when the appropriate matrix environment is provided to maintain relatively higher spatial organization.

**Figure 1:**
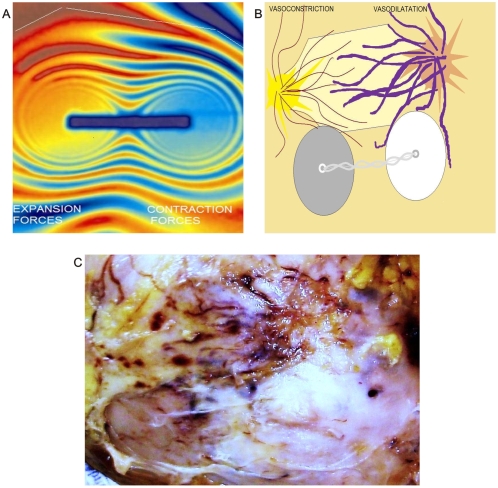
Magnetic field and entangled geometry of a spiral dipole collagen type I vascular framework in cancer tissues. Panel A. The magnetic field of a dipole magnet visualized by spin-polarized neutrons (Credits: Hahn-Meitner-Institute (HMI) I Berlin). Panel B. Spatial organization of image in Panel C. Panel C. Macroscopic dorsal view of renal carcinoma. Collider partners pair of brown and white nodules linked through a fibril collagen bridge. Upper part is structured geometric hexagon pattern constituted by vascular network, on the left are low vascular density with collapse, on the right is seen high vascular density with ecstasia.

Recently, for the first time, researchers from Hahn-Meitner-Institute (HMI) in Berlin [2] have succeeded visualizing magnetic fields inside solid, nontransparent materials through direct 3D images. They used neutrons – subatomic particles having zero net charge – making them ideal for investigating magnetic phenomena in magnetic materials. Neutrons have an internal angular moment, referred to as “spin” in physics, which causes rotation around magnetic fields similar to the way in which the earth rotates on its axis. When all magnetic moments point in the same direction, the neutrons are polarized. If a magnetic sample is irradiated and then collisionate with such neutrons, the magnetic moments of the neutrons begin to rotate around the magnetic fields they encounter in the sample and the spinning direction changes. By detecting spin changes, it is possible to “see” the magnetic field in the sample (Fig. 1A). When comparing their laboratory product image with the images from the present study, the authors detected that both patterns were exceptionally similar (Fig. 1B, 1C). There are statistical universal laws of physics that govern the behavior of magnetic fields. Under this invariant common denominator pattern, one can now apply what is observed and known in other more complex biological or physical systems. Cancer is then revealed as a microcosmos, an excellent model to study chaos both from biological and physical point of view, where collisions, accelerations, and rotational movements generate forms that converge in functions at the interior of the disordered systems. Form is function.

The objective of this article is to uncover a direct dimensional visualization of magnetic field and their observable influence on the collagen vascular behavior inside cancer tissues.

## Materials and Methods

**Figure 2:**
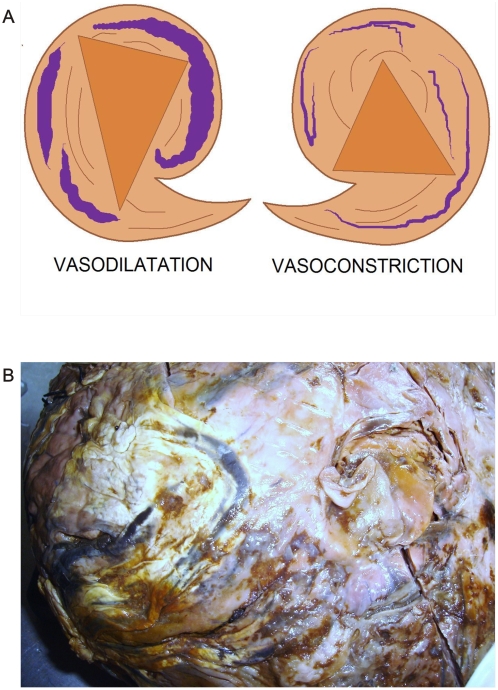
Macroscopic spiral collider partners' dipole vascular behavior. Panel A. Spatial organization of image in Panel B. Panel B. Macroscopic dorsal view of leiomyosarcoma. Collider partners pair of spirals that are oriented in opposed directions. Observe how in the left image the vascular network follows the spiraled pattern with ecstasia and vasodilatation. Image on the right show vasoconstriction. In the center of the spirals appear triangular mirror images on each side.

**Figure 3:**
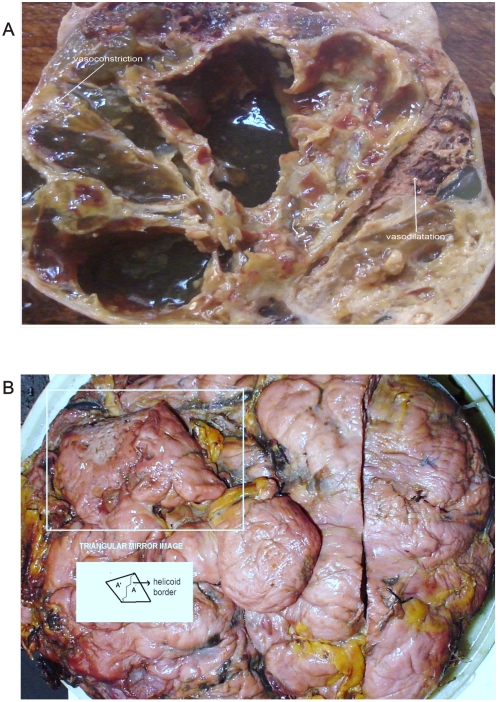
Spatial interrelation between spiral collider partners and GTCHC complexes. Panel A. Macroscopic ventral view of renal carcinoma relate to the same one identified in the Fig. 1 Panel C. Observe dipole biologic tumoral behavior in the proliferative area in spatially opposite position with degenerative cystic changes. Panel B. Macroscopic ventral view of leiomyosarcoma relate to the same one identified in the Fig. 2 Panel B observe triangular mirror images.

**Figure 4:**
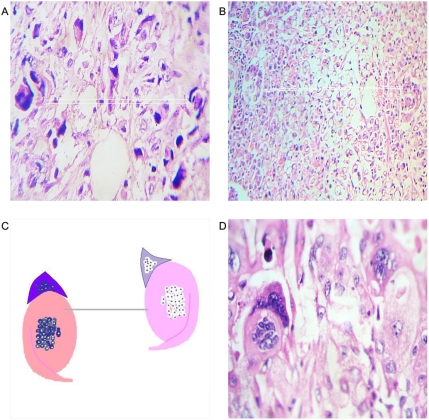
Microscopic collider partners. Panel A. Microscopic view of Breast cancer. Observe pair of spirals orienting in opposite directions. Panel B. Microscopic view of Gastric cancer. Observe pair of spirals orienting in opposite directions. Panel C. Spatial organization of Panel D. Panel D. Microscopic view of Breast cancer. Observe the art-like mosaic mirror images.

In previous observations of GHTCH complexes, we have verified that this organization was based on the causal and sequential activity of collider partner pairs of rotational spiral subpatterns that rotate in simultaneous opposite directions thus originating triangles in inverted position linked one to the other, by helicoidal strings. Reiterative organizations are identified at macroscopic (Figs. 2A, 2B, 3A, 3B) and at microscopic level (Figs 4A, 4B, 4C, 4D) The main concern was to understand how the vascular net of these complexes was organized, what happened behind these complexes in terms of blood supply and organization, and to establish if spiral vascular subpatterns were interrelated with GTCHC complexes assembly inside the tumors. In addition, it is important to prove that GTCHC complexes are not flat geometry but are hierarquic functionally complex geometric attractors.

When pathologists describe a surgical specimen with cancer, they usually give little attention to the disposition of vascular networks, by virtue of a false premise – “Cancer is a complete disorder process.” Pathologists concentrate principally on the ventral areas or cut surfaces, but it is on dorsal serous areas where the vascular net is present. It is in such areas where the vessels penetrate into the tumor.

The vascular net represents the vital nutritional life support of the tumor. On the premise that behind spiral subpatterns of GTCHC complexes a corresponding vascular order must exist, the authors decided to revise rigorously macroscopic and microscopic material of malignant tumors in which GTCHC complexes were identified and coupled with von Willebrand Factor VIII-related antigen analysis. There were a total of 216 old cases and 333 new ones were incorporated. The revision followed the same parameters that were established in the initial phase of the investigation, but with a new item, the visualization and documentation of dorsal serous vascular bed areas in spatial correlation with GTCHC complexes. Samples from histopathology, cythopathology and immunohistochemistry analyses were taken from the respective areas and stained with hematoxylin, eosin, papanicolau, Tricromic and factor VIII antibody.

### Blood Vessels Immunostain

In order to verify the histogenesis of spiral/helicoidal framework related GTCHC complexes, was carried out immunostain label to study distribution, localization and immunoreactivity of von Willebrand Factor VIII-related antigen. 60 formalin-fixed and paraffin embedded tissue sections with the most representative hot spot geometric areas were analyzed. We performed immunohistochemistry using standard protocol method with paraffin sections.[3] Scoring was done as ni, no immunostain; low (10%or less immunopositivity ); high (>10% immunoreactive cells ).

### Electro-Optical Model

#### Experimental design

It is difficult to carry out an appropriate methodological observation for spin-spiraling processes when studying biological systems. But one can obtain indirect information through models from other dynamic systems. What the authors were looking for mainly was to determine if one can reproduce and predict collider partners of spin-spiraled pairs of patterns similar to the ones detected in association with the GTCHC complexes, and if one can reproduce the associated dynamics of expansion, centrifugal light patterns, and centripetal contraction patterns through electromagnetic sequential collisions. In biological terms, it means vasoactive process such as vasoconstriction and vasodilatation behavior, because it is known that vascular cells are influenced by magnetic fields. [4]

To produce such an effect, we have used the standard methodology as in the first publication. [1] The electro-optical model consisted of an electronic flash device attached to a Sony camera model DSC-S600. Strong discharges of light were sent over electric conduction lines (150 V) in a helical pattern. The time intervals were of 3 to 4 min and the light discharges were sent in cycles of 60 min from a distance of 3–4 m in an atmospheric environment and a low temperature of 4°C. To avoid lens flare, the experiment was performed in complete darkness. There were 1-h photographic sessions during a 9-day time interval. To increase the generation and frequency of collider partners of spin-spiraled subpatterns in the collision area, the angle of point of incidence of the light discharge was modified from 45° to 30° over the conductance lines.

### Statistical Analysis

Interrelations between spiral-vascular patterns with GTCH complexes in cancer tissues, and relate factor VIII antibody immunostain patterns was estimated by chi-square for the proportions and carried out with the EPI-INFO 6.04 software.

## Results

### Observations

We have detected macroscopic spatial assembly integration between the spiral vascular assembly in the dorsal serous areas of the tumor (Figs. 1C, 2B) and the ventral cut surface areas where the geometric complexes were located (Figs. 3A, 3B). From the 549 malignant tumors in which GTCHC complexes were detected, direct pairs of spiral vascular mirror assembly were found in 522 cases, that is, in over 95,08% of the cases. Statistical analysis found that for the 549 GTCHC complexes, 522 of these have a vascular spiral pattern become 95,08% of the sample (CI = 92,83%–96,67%) P = 0.000001 and it was negative in 4,91% (27 cases) (CI = 3,32%–7,16%) and P = 0.026. (Table 1)

**Table 1:** Interrelation of Spiral-Vascular Patterns with GTCH Complexes Organization in Cancer Tissues.

GTCHC Complexes	Spiral-Vasc. Patterns+	Spiral-Vasc. Patterns−
549	522	27
549	95,1%	4,9%

**Figure 5:**
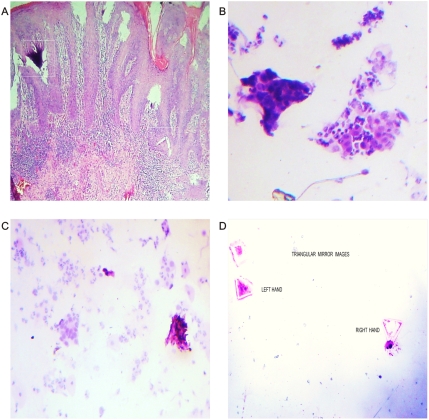
Chromophilic, chromophobic cell affinity-related triangular mirror images. Panel A. Microscopic view of Kaposi's sarcoma. Chromophilic chromophobic triangular mirror images. Panel B. Chromophilic, chromophobic triangular mirror images identified from malignant peritoneal effusion. Panels C, D. Chromophilic, chromophobic triangular mirror images identified from prostate carcinoma smear.

**Figure 6:**
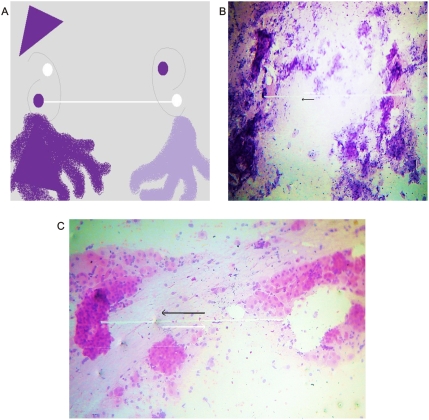
Chromophilic full cell clusters related to chromophobic white empty spaces. Panel A. Spatial organization of Panels B and C. Panels B, C. Microscopic view of collider partners, chromophilic full cell clusters – chromophobic white empty spaces linked through a collagen bridge. Obtained from malignant effusion.

The most striking feature of these findings was to establish that the vessel arranged in asymmetrical patterns had a vasoactive behavior consistent with its spatial location, direction, and sense of spirals. Therefore, we have managed to identify pairs of spiral pattern that rotate in the opposite direction associated with the vascular vasoactive component in the ectasia-vasodilatation or in the collapse-vasoconstriction when turning in the opposite direction (Figs. 2A, 2B) When analyzing vasoactive areas, note that macroscopic vasodilatation corresponded to microscopic chromophilic areas with great affinity to Hematoxylin - Eosin stain, where the tumor is fully active with large number of cell mitosis and pleomorphism. Instead, the macroscopic areas of collapse-vasoconstriction microscopically correspond to cromophobic areas with minimal mitotic activity and apoptotic changes (Figs. 4A, 4B, 4C, 4D; 5A, 5B, 5C, 5D; 6A, 6B, 6C).

**Figure 7:**
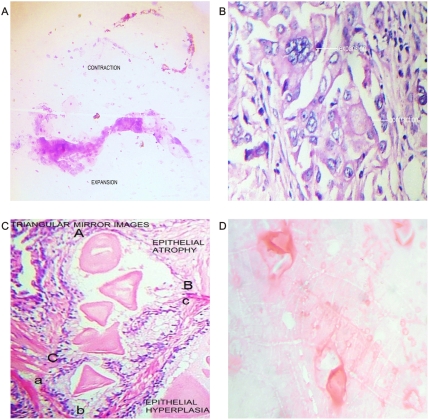
Microscopic chromophilic, chromophobic cell affinity. Related to dipole behavior. Panel A. Contraction and expansion of collider partners from malignant effusion. Panel B. Thyroid cancer cells. Observe triangular mirror image with chromophilic, chromophobic affinity. Panel C. Prostate cancer *in situ*, observe upper crystals with chromophobic affinity, surrounding tissue show epithelial atrophy related vasoconstriction, in mirror position surrounding chromophilic crystals tissue show ephithelial hyperplasia-related vasodilatation. Panel D. Thyroid cancer. Observe triangular mirror image crystals with chromophilic, chromophobic affinity.

The spatial location is arranged so neatly and specifically that it is possible to identify chromophilic nodules as mirror images with white or chromophobe nodules, linked and intertwined through a bridge of collagen fibers (Figs. 6A, 6B, 6C). In addition, the effects of bipolar vessel activity were identified when observing expanding or contracted structures that were always in pairs and as mirror images (Figs. 7A, 7B, 7C, 7D). Clearly, the pairs of spiral vascular arrangement in the serous areas occur in the central magnetic core of the tumor.

**Figure 8:**
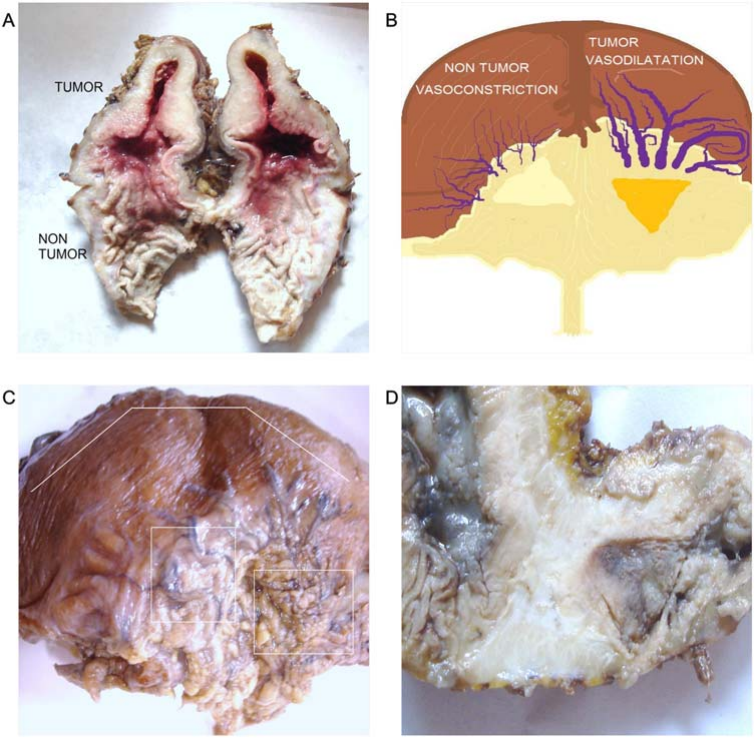
Dipole behavior of Gastric cancer. Panel A. Macroscopic view of cut surface of Gastric cancer despite antro-pyloric tumor restriction. Observe the triangular mirror global-shaped arrangement of the surgical specimen. Panel B. Spatial organization of image in Panel C. Panel C. Macroscopic view of dorsal face image in Panel A. Observe triangular mirror vascular network. On the right is high density vasodilatation, on the left is low density vasoconstriction. Panel D. Macroscopic view of Gastric cancer. Observe triangular mirror image proliferative status adjacent to necrotic cystic degenerative changes.

**Figure 9:**
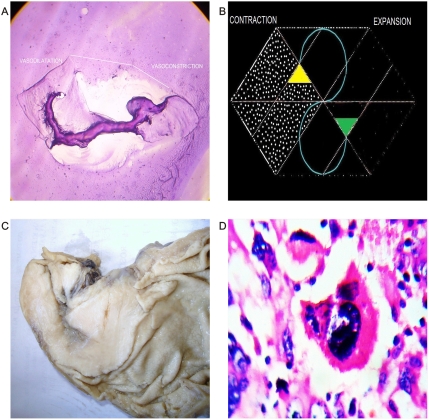
Hierarquic geometric attractor. Panel A. Microscopic view of collagen vascular framework captured from malignant peritoneal effusion. Observe complete development of attractor geometric pattern pairs of triangular mirror images of collagen linked through spiral vasculature orientated in opposite directions. Left section shows vascular density and vasodilatation; right section shows low vascular density and vasoconstriction. Panel B. Schematic design of the geometric attractor identified in the majority of analyzed tumors and the coherent expansion-contraction state generated. Panel C. Macroscopic view of Gastric cancer. Observe collider partner pairs of triangular mirror images linked through a spiral component. Panel D. Microscopic view of geometric attractor in Breast cancer.

**Figure 10:**
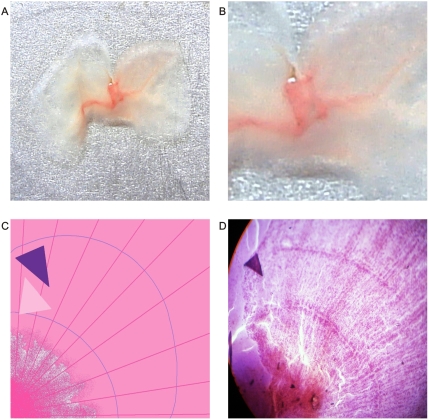
Collagen type I - vascular framework. Panels A, B. Macroscopic view of collagen-vascular framework captured from malignant effusion. Observe triangular mirror image constituted for vasoconstriction–vasodilatation vessels. Panels C, D. Microscopic view of collagen-vascular geometric attractor. Relate to the same one identified in the Fig. 10 Panel B. The right section shows full dynamic activity. Observe two orbits in the inner and outer position that originates from the nucleus. By their rotation and vibration spectral lines emit radial excitation of the cellular components, but the most astonishing characteristic of this image is that the inverted triangular images in each orbit produced by the radial excitations are born in pairs.

**Figure 11:**
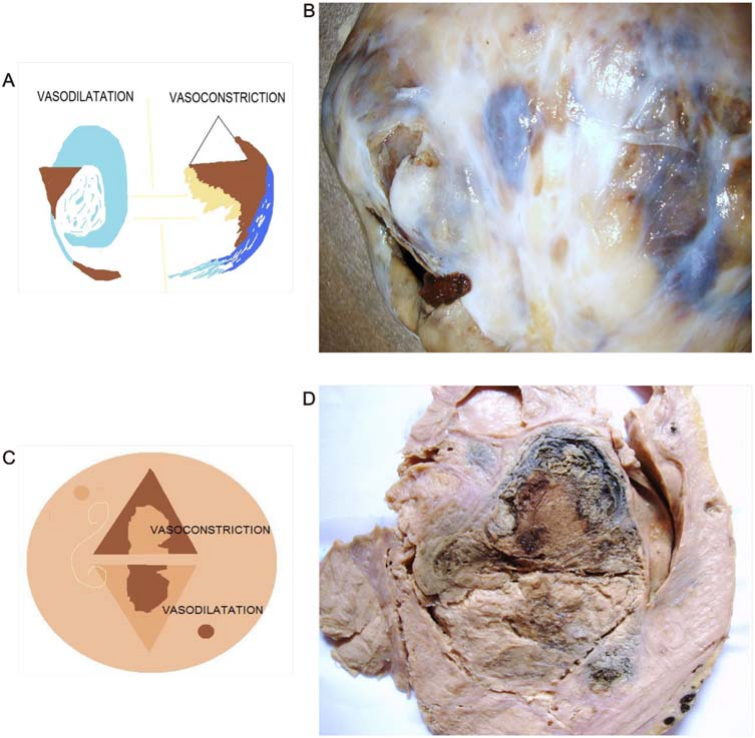
Macroscopic visualization of geometric attractor activity integrating artistic mosaic images in their magnetic domain. Panel A. Schematic spatial organization of image in Panel B. Panel B. Macroscopic serosal dorsal view of renal carcinoma. Observe the elegant geometric attractor mosaic images generated in their magnetic domain. Panel C. Schematic spatial organization of image in Panel D. Panel D. Macroscopic view of the ventral cut surface of Renal carcinoma. Observe the art-like mosaic of triangular mirror images generated at the center of the tumor.

Mirror images of macroscopic and microscopic frameworks of the collagen vascular assembly were identified with observable vasospasm and vasoconstriction activities (Figs. 8A, 8B, 8C, 8D; 9A, 9B, 9C, 9D). In this context, the areas of chromophilic and chromophobic activity are so specific that they imitate a similar mirror image with spatial macroscopic distribution. The organization can be so complex that some form art-like mosaics of perfect images. The assemblies are constituted by collider partners that spin in opposite directions thus developing triangular inverted mirror position images at macroscopic and microscopic levels (Figs. 10A, 10B, 10C, 10D; 11A, 11B, 11C, 11D). At microscopic level, this can implicate the participation of this mechanism in the tumor microvasculature.

**Figure 12:**
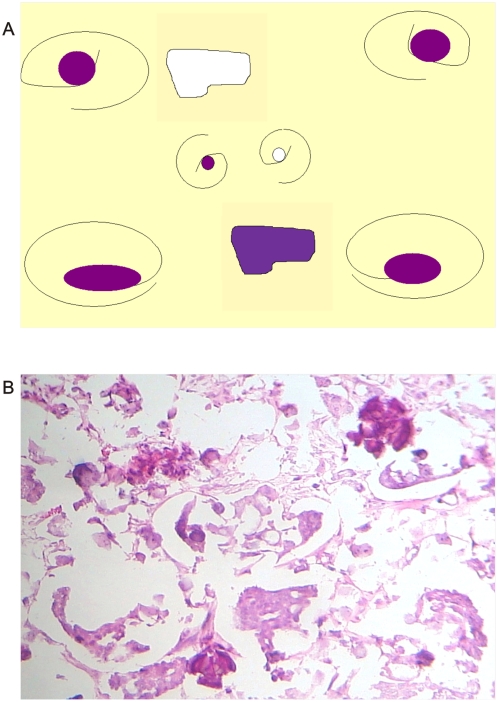
Microscopic art-like mosaic of collider partners mirror images. Panel A. Spatial organization of image in Panel B. Panel B. Microscopic view of malignant Serous Papillary Ovary tumor. Observe the mirror images of the elaborate art-like mosaic of the collider partners. Central pair of spirals with black and white poles are surrounded by corner spatial localization of satellite spiral subpatterns on each side. In the center appear pairs of chromophilic–chromophobic quadrilaterals.

**Figure 13:**
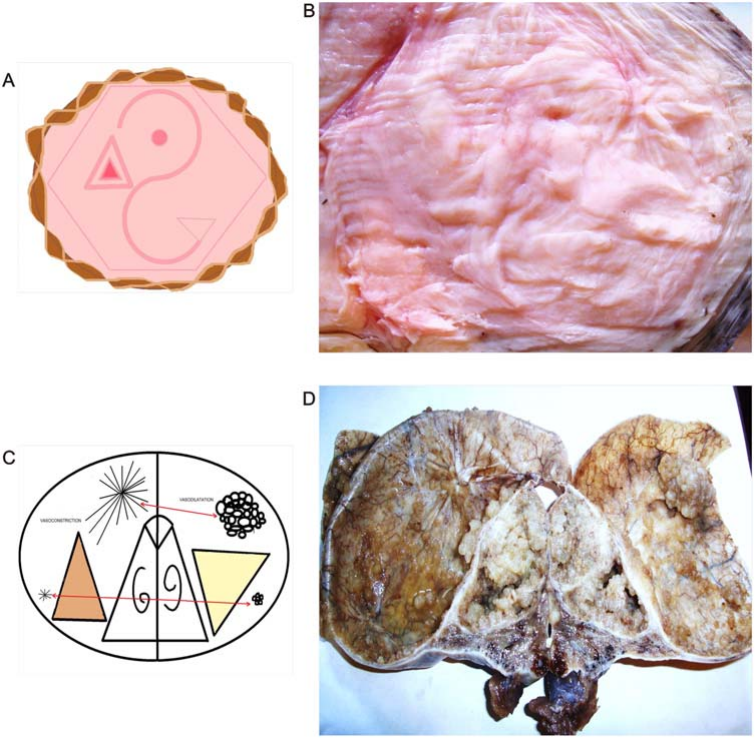
Macroscopic art-like mosaics of geometric attractors. Panel A. Spatial organization of image in Panel B. Panel B. Art-like mosaic of the macroscopic geometric attractor identified in Leiomyosarcoma. Panel C. Spatial organization of image in Panel D. Panel D. Macroscopic view of malignant Serous Papillary Ovary tumor. Observe dipole behavior of collider partners. The left section shows the tumor radial contraction area in relation to triangular cystic pattern. On the right is the proliferative nodule in relation to the inverted triangular solid pattern. In the center are tumor pairs of spirals in opposite orientations.

**Figure 14:**
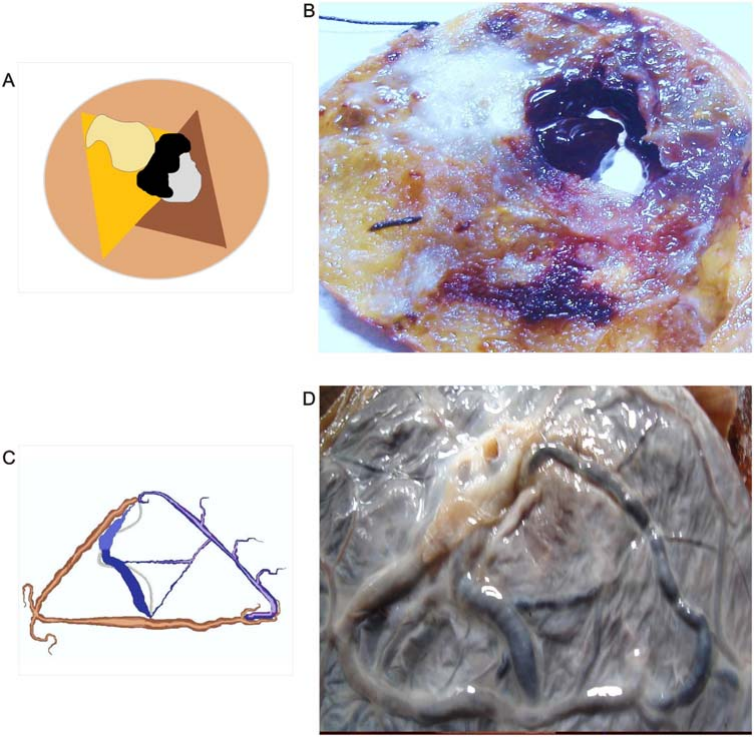
Dipole behavior of collider partners. Panel A. Schematic spatial organization of image in Panel B. Panel B. Macroscopic Thyroid cancer, solid–cystic dipole behavior. Panel C. Schematic spatial organization of image in Panel D. Panel D. Fetal human placenta Chorioangioma. Observe vascular triangular mirror images linked by spiral /helicoidal framework.

When a global analysis of all the macroscopically and microscopically elaborated ordered images is carried out, an invariant morphology of the geometric attractor can be surprisingly identified on the basis of collider partners of triangular mirror images linked by spirals that are expanding and contracting within the system (Figs. 9A, 9B; 10A, 10B, 10C, 10D,). As a consequence, the direct effect on the tumor is to develop a bipolar biologic behavior in terms of proliferative activity and cystic degeneration organized spatially as side-by-side mirror images. The authors' observations repeatedly confirm it (Figs. 12A, 12B; 13A, 13B, 13C, 13D; 14A, 14B, 14C, 14D).

### Blood vessels immunostain

**Figure 15:**
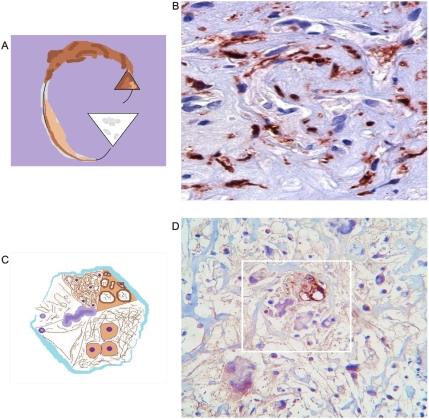
Blood vessels immunostain: Factor VIII antibody. Panel A. Schematic spatial organization of image in Panel B. Panel B. Breast carcinoma. Asymmetric polar activity of factor VIII. Triangular vascular contraction lumen show high immunoreactivity. Triangular mirror vascular dilatation lumen in the opposite pole show complete ausence of the antibody. Panel C. Schematic spatial organization of image in Panel D. Panel D. Malignant Fibrohistiocytoma. Triangular vascular mirror images linked by helicoidal framework. Observe polar asymmetric immunoreactivity.

**Figure 16:**
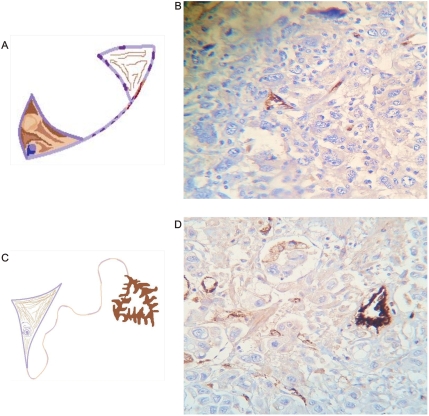
Asymmetric polar Factor VIII immunoreactivity. Panel A. Schematic spatial organization of image in Panel B. Panel B. Lung carcinoma. Triangular vascular mirror images. Observe polar immunoreactivity. Panel C. Schematic spatial organization of image in Panel D. Panel D. Gastric carcinoma Triangular vascular mirror images linked by helicoidal framework. Observe polar immunoreactivity.

**Figure 17:**
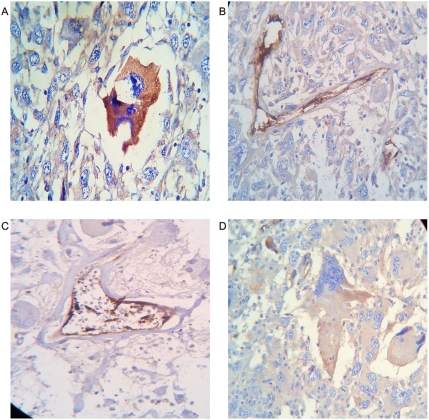
Vascular frameworks polar immunopositivity. Panel A, B. Prostate carcinoma. Observe triangular vascular mirror image, polar immunoreactivity. Panel C, D. Colon carcinoma. Observe Triangular vascular mirror image polar immunoreactivity.

It was evident that Factor VIII related- antigen increase and facilitates the identification of collagen–vascular frameworks in malignant tissues. All 60 tissue labeled sections was positive. In 43 immunostain sections spiral/helical structure poles showed opposed immunoreactive behavior. Polar triangular vascular contraction lumens showed high immunoreactivity, while mirror triangular vascular dilatation lumens in the opposite pole show ausence or low immunopositivity of the antibody. (Figs 15A, 15B, 15C, 15D, 16A, 16B, 16C, 16D, 17A, 17B, 17C, 17D,) Statistical analysis found that for the 60 Geometrical Complexes, 43 of these have a asymmetric polar pattern become 71,6% of the sample P = 0.000002 and it was negative in 28,33%. (Table 2)

**Table 2:** Asymmetric Polar Distribution of Factor VIII Antibody in Spiral/Helicoidal Vascular Framework.

Geometrical Complexes	Asymmetric Polar Pattern	Diffuse Pattern
60	43	17
60	71,6%	28,4%

### Collision event

**Figure 18:**
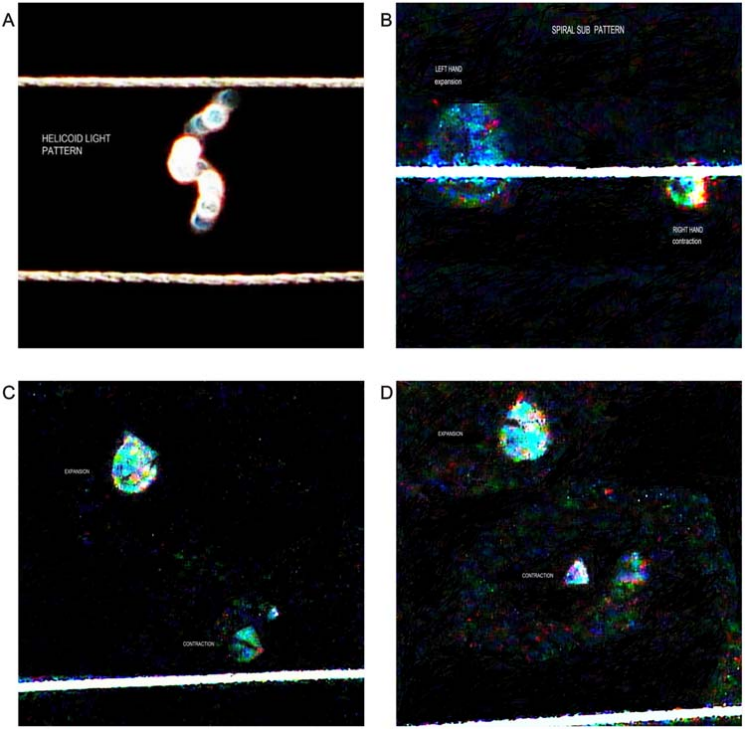
Generation of collider partners from collision in the electro-optical model. Panel A. In the collision interaction region, ejected particles of wave light split into two components that take opposite directions in a helicoid flow pattern with polarization and mirror image. Panel B. Interleaving of subpatterns of indefinite light clusters and defined pairs of left-handed expansion, centrifugal and right-handed contraction, centripetal light spirals are structured in the interaction area. Panels C, D. Pairs of opposite oriented light spirals integrate triangular mirror light clusters to the system. Observe the dipole photodynamic expansion, contraction phases of the geometric triangular patterns.

**Figure 19:**
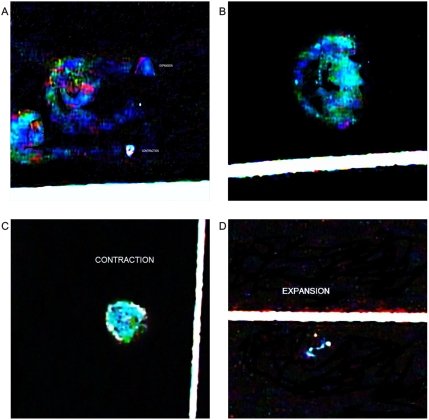
Contraction-expansion phases of collider partners. Panels A, B, C, D. Contraction-expansion phases of triangular light patterns.

In the standard experimental model, the collision of a strong flash from a white light against an electromagnetic field on electronic conduction lines produced the morphodynamics sequential collider partner images. In a dynamic process, at the region of interaction, ejected particles of a light wave split into two components that take opposite directions in helicoidal flow pattern with polarization and mirror images. The luminous energy trajectory is not in a straight line, but follows a helical pattern (Fig. 18A). Triangular and hexagonal light patterns arise on the interleaves of 15–20 subpatterns of indefinite light clusters and on defined left-handed and right-handed spin-spiraled patterns. We have note with great surprise that this rotational behavior is associated with observable centrifugal or expanded disposition of light particles or centripetal contraction depending on the side of the spin (Figs. 18B, 18C, 18D). The triangular patterns originating from these pairs of spiral subpatterns show confluent luminosity (contraction phase) or disperse luminosity (expansion phase). (Figs 19A, 19B, 19C, 19D) show that these spiral patterns were identical to the spiral vascular arrangement in dorsal serous tumor areas.

When comparing the essential pattern found at macroscopic and microscopic levels, the similarity to the one generated through electro-optical collision spiral pairs is surprising. The agreement between the experimental model and real-world tumor biology data images are surprisingly close.

## Discussion

The images presented in this article provide novel information in tumoral biology. The interpretation is that malignant tumors, regardless of the type of tumor, generate geometric attractors amid the biological chaos. This is the intelligent design that nature selects to generate order from disorder. These are not flat geometry; on the contrary, it has surfaces and volumes and is essentially functional. This hierarquic attractor has an invariant morphology of collider partners based on triangular mirror images linked by spirals that represent the interface of molecules that are expanding and contracting within the system, within a specific space-time interval showing both ends of the molecular existence. The life and death of the malignant cells in terms of proliferative and apoptosis status represents the most functional structure sufficient for the flow and delivery of oxygen to tissues for self-repairs. It is interesting to note that this assembly fits exactly with the triangular shape of the lungs that link with the inverted triangular that represents the heart morphology—a trough helicoidal vessel.

Even more interesting is that these patterns have been replicated with great accuracy through the electro-optical model, which indicates a common mechanism generating these geometries and magnetic fields. In making this statement, the authors have the support of recent investigations showing the first 3D images of a magnetic field.

Nikolay Kardjilov's group used this phenomenon as a measurement parameter for tomography experiments using two spin polarizers (which only allow the passage of neutrons whose spin points in a specific direction) to polarize and then analyze the neutrons. By detecting changes in the spins, it is possible to “see” the magnetic fields within the sample.

When comparing this image of a magnetic field in a 3D laboratory with those observed in malignant tissue, the similarity is stunning. (Figs. 1A, 1B, 1C). This fact proves the universality of the physical laws: collider patterns form pairs of dark white nodular areas oriented in opposite directions, linking a trough collagen bridge that results in the dipole functional behavior of the system.

The integration of GTCHC complexes in the ventral and cut surfaces of the tumor with the spiral vascular subpatterns from the dorsal areas results as the visualization of strategic unknown functional volumetric geometric attractor in malignant tissues. What corroborates the idea that the dynamic geometric order occurs through magnetic field activity? As fibers of collagen type I are the largest component of the extracellular matrix (ECM), they are also the biggest generators of electromagnetic activity owing to their piezoelectric ability and conductor behavior. For this reason, mirror images on either side of the midline as well as its poles can be identified (Figs. 6A, 6B, 6C; 7A).

Similar to the manner by which magnetism affects vascular behavior,[4], [5] it influences collagen fibers as well. [6], [7], [8] The vascular network is a series of linked conduits of blood vessels composed of the endothelium, a monolayer of cells that adorn the vessel lumen and surrounding layer(s) of mesenchymal cells (vascular smooth muscle, pericytes and fibroblasts). In addition to providing structural support, the mesenchymal cells are essential for vessel contractility and dilatation. The ECM is a major constituent of blood vessels and provides a framework in which these various cell types are attached and embedded. The composition and organization of vascular ECM is primarily controlled by the mesenchymal cells and is also responsible for the mechanical properties of the vessel wall, forming complex networks of highly regulated structural proteins. The ECM also plays a central role in cellular adhesion, differentiation, and proliferation. The cellular and extracellular matrix components of vessels, with specific emphasis on the regulation of collagen type I, have implications in vascular spatial organization.

The vascular ECM is a complex mixture of collagens, elastin, glycoproteins, and proteoglycans. These constituents not only provide mechanical integrity to the vessel wall but comprise a repertoire of insoluble ligands that can signal the cell to control proliferation, migration, differentiation, and survival. In the normal adult artery wall, the basement membrane is the primary ECM compartment that interacts with the vascular smooth muscle cell (SMC), and its components are believed to be important in maintaining a stable and well-differentiated SMC. However, during conditions of arterial restructuring, the ECM can be quickly remodeled through a combination of synthesis of new ECM molecules, regulated assembly of these molecules, and proteolytic degradation and editing of existing structures. Together, these actions provide a new and dynamic set of ECM stimuli that can have a profound effect on SMC behavior. Some of the major cellular sites for solid-state signaling are “focal adhesions” where integrin receptors mediate the transfer of mechanical force between the cytoskeleton and the ECM. When mechanical forces are applied directly to integrin receptors (e.g., using magnetic forces), cellular biochemistry and gene expression are altered in a stress-dependent manner. Forces applied to integrins activate many signaling pathways in these sites, including protein tyrosine phosphorylation, ion fluxes, cAMP production, and G protein signaling.

In this magnetic domain are attraction and repulsion forces that interact continuously; in physics, attraction between opposite poles is caused by the contraction of ether magnetic particle between the poles. Repulsion between same poles is caused by the expansion of ether magnetic particle between the poles. [9] We were able to replicate, reproduce, and make these forces visible through the electro-optical model (Figs. 15A, 15B, 15C, 15D). One can “see” these forces through the influence exerted by magnetic fields in the global components of the ECM, principally the collagen and the vascular network in cancer tissues. Opposite dipole behavior develops inside the tumor in terms of vasoconstriction and vasodilatation, flow and magnetic field induce collagen vascular alignment, and their magnetic lines finally form self-assembled triangular hexagon GTCHC complexes (Figs. 1B, 1C, 2A, 2B).

The interrelation between the two components is explicit. Collagen conduction in the magnetic field and the vascular driver of fluid in structuring these geometric attractor frameworks habitually tend to treat the two components separately, although they clearly act in coexistence. The conductor (attractive/repulsive and flow) influences the behavior of the fluid and has opposite effects upon the boundaries of the system. It is clear that there exists a paired production behavior in the genesis of these structures. The electro-optical model (Figs. 18A, 18B, 18C, 18D) mirrors what we also found in the pathological cancer tissues (Figs. 2A, 2B, 3A, 3B, 4A, 4B). The literature supports this pairing behavior at different levels: at vascular levels, a multidisciplinary team made up of physicists and biologists from France and Germany* has discovered how, in the embryo, arteries and veins develop in parallel pairs. Using physical measurements, theoretical models, and numerical simulations, the researchers showed how the growth of the arteries directly controls that of the veins through a process that depends solely on the mechanical forces present. [10] This signifies that most of the principal organs embryonically develop in pairs and are in intrinsic relation; during development, what affects one organ would necessarily influence the other paired organ. Pair production is the base of DNA and evolution. During cell division or mitosis, new cells are produced through the growth and division of existing cells. The process begins with the replication of the genetic material held in the chromosomes of the cell. The pairs of sister DNA molecules or chromatids are lined up before being pulled in opposite directions. Partitioning the original cell gives the two new daughter cells the full complement of chromosomes. Physics views pair production as the formation or materialization of two electrons, one negative and the other positive (positron), from a pulse of electromagnetic energy traveling through matter, usually in the vicinity of an atomic nucleus. Pair production is a direct conversion of radiant energy into matter.

Pair production also occurs in tumor biology, and we were fortunate to observe the geometric attractor in full dynamic activity at the macroscopic and microscopic level (Figs. 10A, 10B, 10C, 10D), showing the collagen vascular framework captured from malignant peritoneal effusion. It is possible to see two orbits in the inner and outer positions (Fig. 10C, 10D) originating from the nucleus. During rotation and vibration, the collagens emit spectral lines that are radial excitations of cellular components. However, the most astonishing characteristic of this image is the identification of triangular images born in pairs in inverted positions in each orbit. The two geometric structures originate from the same point; the two images are like twins separated at birth. The communication between these particles is clear, in disorder and in states of anarchy. Communication permits the interchange of information even when particles are not in intimate contact and are far apart. This is possible on the basis of paramagnetism. In physics, paramagnetism signifies that under the influence of an external magnetic field, outer orbital electrons in the atoms of the paramagnetic material will be aligned as a magnet. When the external field is removed, the aligned electrons retain its previous position and the material will lose the magnetism.

This law is the only way to explain the elaborate and structured art-like mosaic images that have been documented (Figs. 11A, 11B, 11C, 11D; 12A, 12B), which means that there is a nexus, that is, an active intercommunication between these geometries that neither needs to be adjacent nor close. From this emerges the vital principle of non-cancer locality, a state in which the entire system is interconnected with other systems but mainly with the processes that are mirror images or in contralateral spatial position. The complex geometries and their constituents share information remotely and generate action. In this fractal geometry, the distance of the particles is irrelevant because the entangled pairs are created. In cancer tissues, hidden variants exist and they become visible only when pairs are created. When pairs of triangles were identified (which were until now hidden structures), they become visible and identifiable structures. In tumor biology behavior, these hidden particles stay in communication no matter how far the particles are. We believe that pair components influence the other component of the pair system even when apart. This communication behavior may come into play by virtue of a strong attraction developing between the colliding partners.

### Dipole behavior

**Figure 20:**
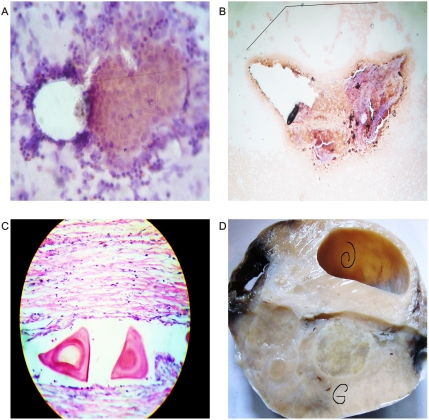
Dipole behavior effect on malignant tissues. Panels A, B, C. Chromophilic full cell clusters related with chromophobic white empty spaces obtained from malignant effusion. Panel D. Macroscopic view of Seminoma tumor. Observe dipole behavior, proliferative nodule in mirror position with cystic degenerative zone.

The images show that the vast majority of malignant tumors have dipole behavior. This was referred to in the previous article. Proliferate areas are in exceptional spatial mirror image position with asymmetric degenerative cystic zones (Figs. 20A, 20B, 20C, 20D). Such polarization is so extreme that it is possible to identify empty cell white nodular areas adjacent or linked through a collagen bridge with full cell nodular proliferative clusters. (Figs. 6A, 6B, 6C) von Willebrand Factor VIII-related antigen characteristics polar immunoreactivity observed permit us determinate the geometric invariant attractor vascular origin and how such structure incorporate dipole behavior to the interior of malignant tissues, this is a consequence of correlated spatial asymmetric assembly of vasoactive vessels that experiment contraction-centripetal or expansion-centrifugal activity in concordance with the spin-spiraled orientation of the molecules. This particular dipole behavior may have great importance in the development of the life of the tumor. In a collision event, bond making reactions that release energy and bond breaking reactions that absorb energy exist. This principle is applicable for both nuclear and chemical reactions. This signifies that mass changes in this process; during expansion, energy is absorbed resulting in an increase of the net masses of the product–mass gains. On the other hand, in the contraction area, the mass of the release matter decreases from the reactants original–mass defect.

This is exactly how the images are explained: mass defect, gains in mirror positions (Fig. 20D). This dipole behavior signifies that each tumor can have vasoconstriction active areas that generate cystic degenerative changes and chromophobic microscopic characteristics related to apoptosis behavior. In mirror spatial position with vasodilatation, more dense vascular chromophilic microscopic areas are found, signifying more active metabolism in relation with more proliferative status.

### Why triangulation?

In this section, we must revert to recent knowledge in physics. From the theory of Causal Dynamics Triangulation (CDT), [11] it is widely accepted that at the very smallest scales space is not static, but is instead dynamically varying. Near the Planck scale, the structure of spacetime itself is constantly changing, because of quantum fluctuations. This theory uses a triangulation process that is dynamically varying and follows deterministic rules, or is dynamical enough to map out how this can evolve into dimensional spaces similar to that of our universe. Physics suggests that this is a good way to model the early universe, and describe its evolution. Using a structure called a simplex; it divides spacetime into tiny triangular sections. A simplex is the generalized form of a triangle, in various dimensions. A 3-simplex is usually called a tetrahedron, and the 4-simplex, which is the basic building block in this theory, is also known as the pentatope, or pentachoron. Each simplex is geometrically flat, but simplices can be ‘glued’ together in a variety of ways to create curved spacetimes. Where previous attempts at triangulation of quantum spaces have produced jumbled universes with far too many dimensions, or minimal universes with too few, CDT avoids this problem by allowing only those configurations where cause precedes any event.

The disadvantageous aspect of this theory is that it relies heavily on computer simulations to generate results. It reveals cancer as a good model to study physics phenomena by virtue of being an environment of multiple collisions and dynamic fluctuations.

In the investigation, we have documented hundreds of triangular mirror images. It is a reality that has been extensively documented, and its frequency is directly proportional to the degree of tumor aggressiveness. Form is function: under this premise, in tumor biology, one can say that triangular geometric pattern represents for less the way to reorganize disturbed systems. We have outline the hypothesis to open the discussion in light of verifiable and reproducible facts on each patient who suffers from cancer.

### Some questions

**Figure 21:**
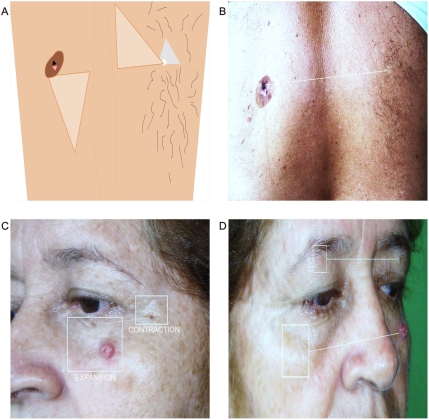
Visualization of geometric attractor activity. Outside malignant tumors. Panel A. Schematic spatial organization of image in Panel B. Panel B. Shows Melanoma tumor. Observe on left the circular black lesion and hair loss which are integrated on the right contralateral “healthy” zone with a mirror triangular attractor, where a white lesion can be identified. High hair density lay in a helicoidal pattern. Panels C, D. Shows Squamous cancer cell tumor. Observe how in exactly “Healthy” contralateral position a clearly discernable lesion appears.

After conducting this physics and biological approximation to explain the dynamics of the complex GTCHC, a reasonable doubt emerges: what happens at the macroscopic, microscopic, and molecular levels in the contralateral *outside* of a tumor primarily those arising in paired organs? This article shows that malignant tumors generate collider partner images in their magnetic domain with opposite biological behavior *inside* and *outside* of malignant tumors (Figs 21A, 21B, 21C, 21D). These figures represent excellent demonstrations of such affirmations. The first image shows a melanoma, one of the most aggressive tumors in human tissues. Observe at the left side a circular, black lesion of hair loss that integrates to the right side contralateral “healthy” zone with a mirror triangular attractor, where a white lesion and high hair density in helicoidal pattern can be identified. The second image shows squamous cancer cell tumor. Observe how a lesion is clearly discernible in a “healthy” contralateral position.

These images evoke a dipole biological behavior – inside and outside – in relation to the spatially opposite tumor location. From this point of view, we have considered it fundamental that the single monopolar proliferative actual view of cancer must be replaced by a demonstrated dipole biologic behavior.

**Figure 22:**
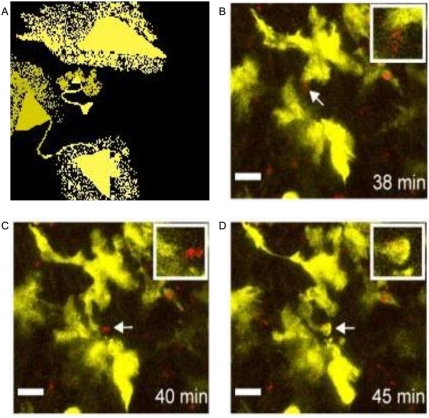
Geometric attractor invariant morphology identified in inner workings of the immune system. Panel A. Schematic spatial organization of images in Panel BCD. Panel B, C, D. Dendritic cells structuring triangular mirror images with extension/retraction of their pseudopods in spiral/helicoidal pattern to engulf protozoan parasites. Observe asymmetric polar photoluminescence. Multi-photon microscopy Image (Credits: Australia, Sydney's Centenary Institute Immune Imaging program. Inner Workings of The Immune System 2008.

**Figure 23:**
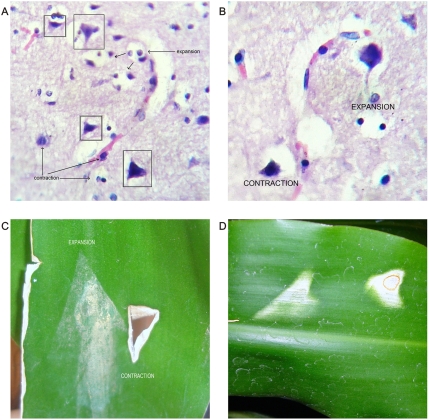
Geometric attractor invariant morphology identified in different biologically disturbed systems. Panels A, B. Microscopic Brain cortex from deceased cancer patient. Observe how pyramidal cells are assembled in mirror position around the vessel. At each side neuroglial cells adopt opposite expansion–contraction behavior. Panels C, D. Fungal plant pathology. Observe invariant morphology of geometric attractor.

**Figure 24:**
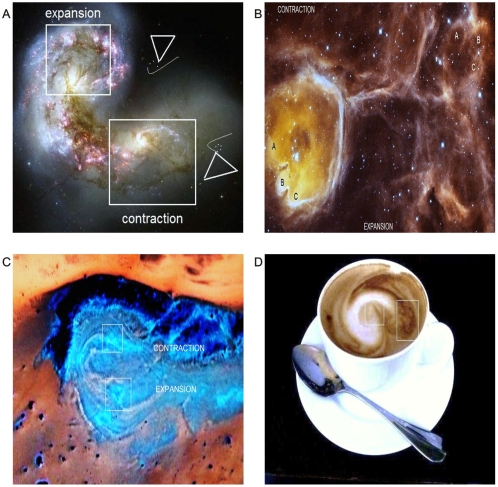
Visualization of geometric attractor in Collision systems. Panel A, B. Galaxy collision. Observe the hierarquic geometric attractor (Credits: NASA Hubble images). Panel B. Tectonic plate collision. Observe the hierarquic geometric attractor (Satellite image). Panel C. This hierarquic attractor is present in the daily breakfast coffee.

Recapitulating the findings of the first and actual article, we have affirmed that collision events in sequential stages identified in cancerous tissues are: Generation of collider partners – particles that spin in opposite directions. In synchronic and simultaneous movement in the presence of a magnetic field, the electrons may point ‘left’ or ‘right’. This rotation generates powerful magnetic field lines. The electron particles are aligned in their domain, and the flux in the magnetic lines are distributed in the ECM along the collagen and vascular components. Because electrons flow spirally through a conductor (collagen type I), these magnetic lines move with the spiral. Pair production, inherent pair development and dependence, induces the flux to flow in the opposite direction. In this manner, particles expand or go away, and others come closer or are contracted from a fixed point. This entangled phenomena produce dipole behavior in terms of metabolic proliferation and apoptosis status, a process that is revealed in multiple outbursts of biological collisions in malignant tumors. This complex is fractal always evolving into more complex geometric *hexagonal* structures over time-space intervals. It is a predictable and reproducible dynamic system. It is identified on systems in states of disorder regardless of the type of system involved. It has an invariant morphology with sets of triangular mirror images in opposite positions and spiral-patterned visible attractors. The hierarquic geometric attractor is important not only in human biologic tissues such as tumors but also in physics and is so *universal* that we have identified this invariant pattern for the first time in other perturbed systems: Inner workings of the immune system. [12] (Fig. 22A, 22B, 22C, 22D) Brain cortex patients deceased from cancer (Figs. 23A, 23B); in plant fungal pathology (Figs. 23C, 23D); Galaxy collisions (Figs. 24A, 24B); Geological collision tectonic plates (Fig. 24C); and, in fact, routine and simple that when coffee and milk are mixed for breakfast, this attractor is present (Fig. 24D).

Theoretical models of generic networks have revealed that stable states known as high dimensional “attractors” self-organize in large interconnected networks containing thousands of elements, if they exhibit a particular class of network architecture. Virtually all biomolecular networks analyzed to date have this architecture. Stable, high-dimensional attractor states arise from the system level as a consequence of particular regulatory interactions between the network components (e.g., genes) that impose constraints on the global dynamics of the network; thus, the cell cannot occupy any arbitrary network state. On these theoretical grounds it is proposed that different cell types (e.g., lung vs. heart) represent different attractor states in the gene regulatory network. It is important because it explains how cells can simultaneously sense multiple chemical, adhesive, and mechanical inputs and yet only switch on one of a limited number of specific, reproducible behavioral responses. This may explain how global changes in shape are able to control cell-fate switching.

For this reason, structure dictates function in living cells – cells can be switched between growth, differentiation, and death solely by varying the degree to which it physically distorts its shape. [13], [14].

Although the authors believe that geometry can control cells from growth to apoptosis through the macropatterned and micropatterned attractor collagen type I vascular framework of the ECM, the viability of the influence of magnetic fields substrates may represent a fundamental mechanism for the development of proliferative regulation within the tissue microenvironment.

At this point the findings suggest that in tumoral biology systems, geometric attractor patterns constituted collider partners, emerging when a steady equilibrium state is disturbed. These attractor patterns probably govern how molecules self-assemble from *de novo* holding a vision of a new renaissance tissue in a coherent expansion-contraction state.

In conjunction with the dimensional organization of intelligent functional geometry [15] it is determined that an invariant single common denominator pattern can drive multiple change scenarios that emit the precisely shaped signals necessary to incorporate notions of location control, a trough in the vasoconstriction and vasodilatation inside the system. This implicates the importance of position and location that cells have in cancer development [16], [17], [18], [19]. If it is possible to incorporate these principles (geometric invariant attractor) into artificial nanomaterials, biomedical devices, engineered tissues, or become the structural template for rationally designed drugs one could develop *new therapeutic* cancer strategies.
